# Detecting visual texture patterns in binary sequences through pattern features

**DOI:** 10.1167/jov.23.13.1

**Published:** 2023-11-01

**Authors:** Maria F. Dal Martello, Keiji Ota, Dana E. Pietralla, Laurence T. Maloney

**Affiliations:** 1 Dipartmento di Psicologia Generale, Università di Padova, Padova, Italy; 2 Department of Psychology, New York University, New York, NY, USA; 3 Center for Neural Science, New York University, New York, NY, USA; 4 Institute of Cognitive Neuroscience, University College London, London, UK; 5 Department of Psychology, University of Cologne, Cologne, Germany

**Keywords:** pattern perception, texture perception, cue combination, feature combination, Markov process, binary sequences, hot hand, gambler's fallacy, Bayesian decision theory, signal detection theory, randomness, Kolmogorov–Chaitin complexity

## Abstract

We measured human ability to detect texture patterns in a signal detection task. Observers viewed sequences of 20 blue or yellow tokens placed horizontally in a row. They attempted to discriminate sequences generated by a random generator (“a fair coin”) from sequences produced by a disrupted Markov sequence (DMS) generator. The DMSs were generated in two stages: first a sequence was generated using a Markov chain with probability, *p_r_* = 0.9, that a token would be the same color as the token to its left. The Markov sequence was then disrupted by flipping each token from blue to yellow or vice versa with probability, *p_d_—*the probability of disruption. Disruption played the role of noise in signal detection terms. We can frame what observers are asked to do as detecting Markov texture patterns disrupted by noise. The experiment included three conditions differing in *p_d_* (0.1, 0.2, 0.3). Ninety-two observers participated, each in only one condition. Overall, human observers’ sensitivities to texture patterns (*d′* values) were markedly less than those of an optimal Bayesian observer. We considered the possibility that observers based their judgments not on the entire texture sequence but on specific features of the sequences such as the length of the longest repeating subsequence. We compared human performance with that of multiple optimal Bayesian classifiers based on such features. We identify the single- and multiple-feature models that best match the performance of observers across conditions and develop a pattern feature pool model for the signal detection task considered.

## Introduction

Observers respond to illusory patterns in sequences of events, exhibiting “sequential effects” in many tasks: in decision-making (Ayton & Fischer, 2004; Barron & Leider, 2010; Kahneman & Tversky, 1972; Roney & Trick, 2009), perception (Howarth & Bulmer, 1956; Maloney, Dal Martello, Sahm, & Spillman, 2005; Speeth & Mathews, 1961), motor behavior (Cho et al., 2002; Soetens, Boer, & Hueting, 1985), and prediction (Schüür, Tam, & Maloney, 2013). Electroencephalographic and functional magnetic resonance imaging responses show sequential effects (Leopold, Wilke, Maier, & Logothetis, 2002; Squires, Wickens, Squires, & Donchin, 1976). Previous studies have found sequential effects in two-alternative forced-choice reaction times tasks (Remington, 1969) even after more than 4,000 trials (Soetens et al., 1985). Basketball spectators and players often believe that players experience extended periods of elevated probability of success, although statistical analysis finds no such “hot hand“ patterns (Gilovich, Vallone & Tversky, 1985; Tversky & Gilovich, 1989a, Tversky & Gilovich, 1989b). The hot hand is not limited to basketball. The literature on similar phenomena is considerable (Bar-Eli, Avugos & Raab, 2006); nonhuman primates can show a hot hand bias (Blanchard, Wilke & Hayden, 2014).

A different pattern—the gambler's fallacy—emerges when individuals judge a failure more likely than an additional success after a series of successes or vice versa (Gilovich, 1991; Tversky & Kahneman, 1971; Tversky & Kahneman, 1974). Human observers are said to be overly sensitive to possible patterns, finding illusory connections between events (Conrad, 1958; Foster & Kokko, 2009; Maloney et al., 2005; Mishara, 2010; Shermer, 2008). Observers find deviations from randomness in binary sequences where none are present.

But, when a failure of randomness (a pattern) of some kind is actually present, how successful are observers at detecting it? If, for example, a roulette wheel is actually biased in favor of red, can the gambler detect the failure of randomness and make use of it, betting exclusively on red? How do they go about deciding that a sequence is not random?

**Figure 1.**
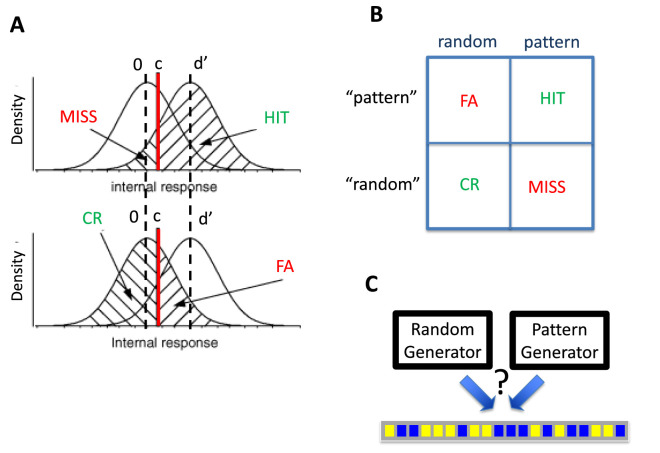
Detecting patterns. Following (Lopes, 1982; Lopes & Oden, 1987) we model discrimination of patterned and random sequences as equal-variance Gaussian signal detection theory (SDT). (**A**) The SDT model. A sensory evidence variable is compared to a sensory criterion *c.* The observer responds “pattern” if the evidence variable exceeds the sensory criterion, otherwise “random.” (**B**) Possible outcomes The possible outcomes (HIT, MISS, FALSE ALARM [FA], CORRECT REJECTION [CR]) are shown. In SDT terminology, a tendency to find illusory patterns corresponds with a high FA rate. (**C**). The SDT task. One of two generators is chosen at random. The observer—given the generated sequence—attempts to identify the generator used. We emphasize that the observer is asked to identify the generator that gave rise to the sequence—an objective task—not to judge the intrinsic or subjective randomness of the sequence.

There is clearly a tradeoff between “hallucinating” a nonexistent pattern and overlooking an actual deviation from randomness. The tradeoff between “hallucination” and detection is made obvious when it is framed as a YES–NO signal detection task (Green & Swets, 1966/1974) (Figure 1A, B) where the signal is the presence of a pattern in a sequence. Signal detection theory (SDT) (Green & Swets, 1966/1974) is a special case of Bayesian decision theory (Maloney & Zhang, 2010). The observer views a sequence generated by one of two generators chosen at random with equal probability (Figure 1C) and judges whether the sequence was produced by the random generator or by the pattern generator. The generators are defined elsewhere in this article. The observer's sensitivity to pattern is measured by the signal detection parameter *d′*, but this measure alone does not determine the observer's response. In normative equal-variance Gaussian SDT, the response is also affected by choice of a sensory criterion *c* (Figure 1A).

The four possible outcomes of a signal detection trial are HIT, MISS, FALSE ALARM (FA), and CORRECT REJECTION (CR) (Green & Swets, 1966/1974; Figure 1A, B). An FA occurs when the signal is not present, but the observer responds “yes,” analogous to hallucinating a pattern. The observer can reduce the FA rate by moving the sensory criterion rightward, reducing both the FA rate and the HIT rate. In normative SDT, criterion *c* is set to maximize expected gain given the penalties and rewards associated with each outcome and the prior probability that a signal is present.

(Lopes, 1982; Lopes & Oden, 1987) first reformulated the problem of pattern detection within the framework of SDT. Lopes & Oden asked observers to discriminate binary patterns generated by Markov chains (defined below) from binary patterns generated by a “fair coin.” We consider a wider class of possible patterns, disrupted Markov sequences (DMS)—Markov sequences disrupted by noise. Use of DMSs will allow us to assess what information observers use in making their decisions.

### Random and pattern generators

**Figure 2.**
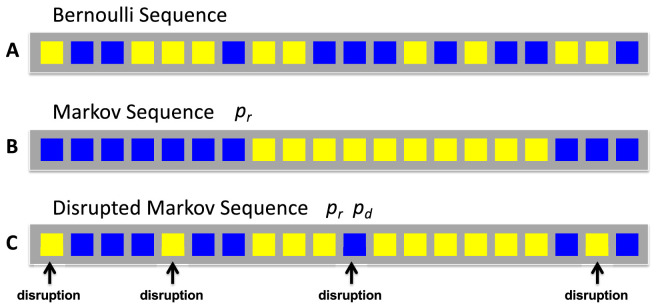
Stimulus generators. (**A**) A random generator. Each token is equally likely to be blue or yellow and the choices of colors across the sequence are statistically independent. We refer to the generating process as “random.” (**B**) A Markov generator. The leftmost token is equally likely to be blue or yellow and each token going from left to right has probability *p_r_* (the probability of repetition) of being the same color as its predecessor. If 0.5 < *p_r_* < 1 the resulting sequence will tend to have monochromatic streaks. In this example, *p_r_* = 0.9. (**C**) A disrupted Markov generator. To generate a disrupted Markov sequence, we first generate a Markov sequence and then independently “flip” each token in the sequence from blue to yellow or vice versa with probability *p_d_*, the probability of disruption. During the main experiment observers saw only random sequences and disrupted Markov sequences, not undisrupted Markov sequences. We include Figure 1B to aid in explaining Figure 1C; we used such Markov sequences only during training.

On each trial in the experimental task, observers viewed a sequence of 20 simultaneously presented colored tokens (Figure 2A) and judged whether the sequence was generated by one of two generators: the random generator or the patterned (DMS) generator. We refer to these sequences as “patterns,” “textures,” or “texture patterns” interchangeably. We presented sequences simultaneously rather than sequentially to minimize any effect of memory limitations on human judgment.

We first explained to observers how patterned and random sequences were generated and told them that sequences were as likely to be generated by the random generator as by the pattern generator on each trial. The signal detection task was to identify the generator on each trial given only the generated sequence. The sequences were generated anew on every trial.

Random sequences (Figure 2A) were generated as independent, identically distributed Bernoulli random variables with probability 0.5 of being blue and otherwise yellow. Both generators are stochastic processes and the sequences generated by either could correctly be described as random. In our explanation of the task to observers we always used the terms “random” and “pattern” or “patterned” as labels to describe the sequences generated by the two generators and the generators themselves. We use these terms here as well. When convenient, we will treat the sequences as sequences of binary digits with 1 interpreted as blue, 0 interpreted as yellow.

Patterned sequences (DMSs) were generated by first generating a Markov sequence (Figure 2B) and then disrupting it. The binary Markov chains we used were characterized by a single parameter *p_r_*, the probability that each successive token, left to right, would be the same color as the preceding. The leftmost token—lacking a predecessor—was as equally likely to be blue as yellow. In Figure 2B, *p_r_* = 0.9, and the sequence tends to alternate less often than a random sequence.

The Markov sequence was then disrupted. With probability *p_d_*, we independently flipped each of the tokens in the Markov sequence from blue to yellow or vice versa (Figure 2C). If *p_d_* is 0, the resulting DMS is just a Markov sequence similar to those used by (Lopes, 1982; Lopes & Oden, 1987). If *p_d_* is 0.5, the resulting DMS is effectively a random sequence: the disruption process with *p_d_* equal to 0.5 removes any pattern in the disrupted sequence.

Binary sequences are particularly appropriate for our purposes as people “often reframe events as binary sequences occurring over time” (Oskarsson, Van Boven, McClelland & Hastie, 2009, p. 262). Markov chains have been used to model human cognitive learning processes (Atkinson, Bower & Crothers, 1965; Bush & Mosteller, 1955; Greeno, 1974) and multiperson interactions (Suppes & Atkinson, 1960). Much of the literature concerning human perception of patterns just described uses such random sequences, sequences of successes and failures, or even just heads and tails. The task is effectively texture discrimination between two families of one-dimensional binary textures (Landy & Graham, 2004).

By varying the Markov parameter *p_r_* we can generate sequences for which the Gambler's fallacy is not a fallacy (*p_r_* < 0.5) and the hand can be—from time to time—truly hot (*p_r_* > 0.5). The level of disruption *p_d_* affects the difficulty of the task and will help us in investigating how human observers carry out the task and in discriminating between candidate models of human performance.

We emphasize that the observer was asked to judge whether a particular sequence was *generated* by the random generator or the patterned (DMS) generator, not to judge whether the observed sequence was intrinsically random or patterned. There is a rich literature (Bar-Hillel & Wagenaar, 1991; Tversky & Kahneman, 1971; Tversky & Kahneman, 1974; Wagenaar, 1970a; Wagenaar, 1970b; Wagenaar, 1972) on observers’ judgments of the intrinsic randomness of binary sequences. Given two sequences produced by independent tosses of a fair coin such as HHHHTTTT and HTTHTHTH, the first sequence is judged to be less random or less probable by many, even though they have equal probability of occurrence. Parallel work by Solomonoff, Chaitin, and Kolmogorov permits assigning an objective intrinsic randomness to any sequence (Chaitin, 1987; Kolmogorov, 1963; Kolmogorov, 1965; Solomonoff, 1960; see Li & Vitányi, 2019 for a review). By framing the task in SDT terms, we obviate any need to decide whether patterns are truly random or truly patterned. Even so, our results may prove relevant to determining the features of texture sequences that lead human observers to classify them as intrinsically random or nonrandom. We return to this point in the Conclusion.

## Methods and materials

### Stimuli

There were three experimental conditions, each 100 trials long, each consisting of 50 random texture sequences and 50 patterned texture (DMS) sequences. The 100 sequences were presented to the observers in randomized order. Each sequence—presented individually on a computer screen—comprised 20 tokens. The tokens were of the same size and shape and could be either yellow or blue. The tokens were arranged, one after the other, in a horizontal line. Examples are shown in Figure 2A (random) and Figure 2C (patterned: DMS). Sequences were newly generated on every trial.

We refer to the three conditions as low disruption (LD), medium disruption (MD), and high disruption (HD). The conditions differed in the probabilities of disruption *p_d_*, which was 0.1 in LD, 0.2 in MD, and 0.3 in HD. The repetition probability *p_r_* used in generating all the DMS sequences was *p_r_* = 0.9 for all conditions.

### Observers

Ninety-six observers were recruited in the common areas of the University of Padova. Four were excluded because their responses were ambiguous or indicated they had not correctly understood the task. Of the remaining 92 observers, 33 (17 females, 16 males) were in condition LD, 19 (11 females, 8 males) in condition MD, and 40 (21 females, 19 males) in condition HD. The observers' median age was 23 (range, 18−34 years). We included extra observers in the extreme conditions to increase statistical power.

### Procedure

The experiment was conducted in a large computer classroom at the University of Padova. There were two experimenters and one or more observers present in the classroom during the experiment. Each observer was assigned, in a between-subjects study design, to just one of the three conditions. The instructions and the stimuli were presented on a computer screen using Microsoft Power Point. The observers read the instructions and made responses at their own pace. The responses were given by marking a form. The experiment was structured in three stages: instructions, training and main experiment.

#### Instructions

In the first stage, the observers read through an explanation of the generating processes for random sequences and Markov sequences. Then they were shown five labeled examples of each. Next we explained in detail how we disrupted the Markov sequences and presented three examples contrasting a Markov sequence with two DMSs with different levels of disruption. All observers in all conditions received exactly the same instructions. An experimenter was available to answer any questions the observer might have. We did not use the term DMS with observers but rather the terms “pattern“ or “patterned.”

#### Training

The observer practiced the task in their condition (LD, MD, or HD). Twelve sequences, one-half random and one-half DMS, were presented in random order and observers judged whether the sequence viewed was generated by the random process or the DMS process for their condition. They received correct feedback.

#### Main experiment

In the main experiment, the observers saw 100 sequences, one-half random and one-half DMS, presented in random order. As in training, they had to judge whether each sequence was random or patterned. Unlike in training, feedback was not given. The time for responding was at the observer's discretion. At the end of the experiment, the observers were asked to explain in writing any strategies they believed that they used to carry out the task. See the Supplement for a summary and analysis of their responses.

### Models and model fitting

#### Full BDT model

We first compared human performance to that of a Bayesian decision-theoretic (BDT) model maximizing the probability of a correct response. It is implausible that any human observer would be perfectly Bayesian (Maloney & Mamassian, 2009), but Bayesian performance is an upper bound on possible human performance. Comparing ideal performance to actual performance has proven to be fruitful in many areas of research (Geisler, 1989; Maloney & Zhang, 2010; Trommershäuser et al., 2003a; Trommershäuser et al., 2003b; Trommershäuser et al., 2008).

The full BDT model and fitting procedure coincide with those used for equal-variance Gaussian SDT, which is a form of Bayesian decision theory (Green & Swets, 1966/1974). We computed the posterior odds that a sequence *S* was produced by the DMS generator or the random generator,

(1) $$\begin{array}{ccc} \lambda \; & = & \frac{P[DMS|S]}{P[RANDOM|S]}\\ & = & \frac{P[S|DMS]}{P[S|RANDOM]}\frac{P[DMS]}{P[RANDOM]}\\ & = & \frac{P[S|DMS]}{P[S|RANDOM]} \end{array}$$

as prior odds $\frac{P[DMS]}{P[RANDOM]}=1$ by design (Maloney & Zhang, 2010). We interpret a larger value of λ as evidence favoring DMS over Random. The normative classification rule is to respond patterned (DMS) whenever λ > 1. In most applications of SDT the judgment rule used is “respond patterned if and only if λ > *L*” and the likelihood ratio criterion *L* is estimated from data. Here, however, we are considering an ideal Bayesian rule that maximizes the proportion of correct responses, and for such as rule *L* = 1 necessarily as the prior odds (random or patterned) are 1. In effect we compute the probability that the sequence came from the RANDOM generator and the probability that it came from the PATTERN generator and choose whichever generator is more probable given the sequence. The details of computing λ are described in the Appendix.

#### Single- and multifeature models

**Figure 3.**
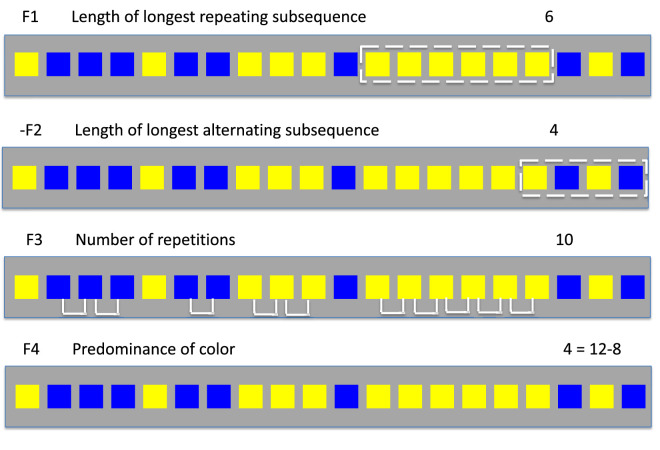
Candidate feature models. Following the experiment, observers were asked to describe how they distinguished between stimuli generated by the random generator and by the disrupted Markov sequence (DMS) generator. These responses are summarized in Supplement 1 and were reduced down to candidate features. We do not assume that a feature mentioned by one or more observers is actually used by those observers—or any observers—in carrying out the task. The analyses of observers’ reports simply give us a list of candidate features to evaluate. Each feature is a computation on the sequence the observer saw, for example, the length of the longest sequence of a single color. Four of those features are illustrated here. For three of the features (F1, F3, and F4) a larger value is evidence that the sequence is DMS, not random. For one of the features (F2), larger values are evidence in favor of random over DMS. In analyses we simply multiply this feature value by –1, using –F2 as the feature value.

We define a feature as any function *φ*(*S*) applied to a sequence *S* that returns a number, for example, the “length of the longest repeating subsequence” in the sequence *S*. Our choice of potential features is defined in Figure 3. These features were chosen based on observers’ own reports of how they carried out the task (see the Supplement). We do not assume observers are aware of the features they use in carrying out the task or that they used the features they claimed they used. We eliminated features whose use is not supported by the data.

The full BDT model has access to the sequence S itself and, thereby, to all possible features. The feature models mimic observers who choose to use only a portion of the information available—one or more features. The observer may base his judgment on a single feature or possibly combine two or more features.

The BDT decision rule based on *K* features would be

(2) $$\begin{array}{c} \;\;\;\;\;\text{Respond}\;\text{PATTERN}\;\text{(DMS)}\;\text{if}\;\text{and}\;\text{only}\;\text{if}\;\lambda >1\;\; \end{array}$$

with

(3) $$\begin{array}{c} \lambda =\frac{P\left[DMS|\varphi_{1}\left(S\right),\cdots ,\varphi_{K}\left(S\right)\right]}{P\left[\mathrm{Random}|\varphi_{1}\left(S\right),\cdots ,\varphi_{\kappa }\left(S\right)\right]}.\; \end{array}$$

Intuitively, the multifeature models mimic an observer who chooses to use a portion of the information available based on one or more features, but is otherwise normative Bayesian. This rule maximizes the expected probability of a correct response.

A drawback of the multifeature models defined in this way is they permit arbitrary interactions among the features: if a single feature value increases, there is no guarantee that the overall decision variable λ increases. If we wish to interpret individual features as evidence against randomness and in favor of patterns, we must restrict how they interact.

Accordingly, we adopt a simpler approach based on a generalized linear model (GLM) (Dobson, 2002; McCullagh & Nelder, 1989). The resulting model is analogous to weighted additive cue combination (Boyaci, Doerschner & Maloney, 2006; Landy, Maloney, Johnston & Young, 1995; Landy, Maloney, Young, 1991). The GLM selects weights β_*k*_,*k* = 1, ⋅⋅⋅, *K* and estimates

(4) $$\begin{array}{ccc} & & E[P[DMS|S]]\\ & & \;=\psi (\beta_{0}+\beta_{1}\varphi_{1}\left(S\right)+\cdots +\beta_{K}\varphi_{K}\left(S\right))\; \end{array}$$

where *E*[ ] denotes mathematical expectation and ψ( ) is the logistic function

(5) $$\begin{array}{c} \psi \left(x\right)=\frac{1}{1+e^{-\left(x-x_{0}\right)}}.\; \end{array}$$

The GLM analysis selects weights *β_k_* that represent the contribution of each feature ψ_k_(*S*) to the decision. The features may be highly correlated (it is likely they are), but the GLM analysis compensates for any correlation. We base our analyses on these weights. We compare human performance with performance predicted by the full model, by GLM models based on single features, and by the GLM models based on two or more features.

The performance of any feature model—single-feature or multifeature—is bounded above by that of the full model. Our goal is to find the feature model based on one or more features that best matches the choices of each human observer—if there is one. We used observers’ self reports to define a set of candidate features that might account for human performance. We compare the performance of the resulting “candidate“ feature models to human performance.

## Results

**Figure 4.**
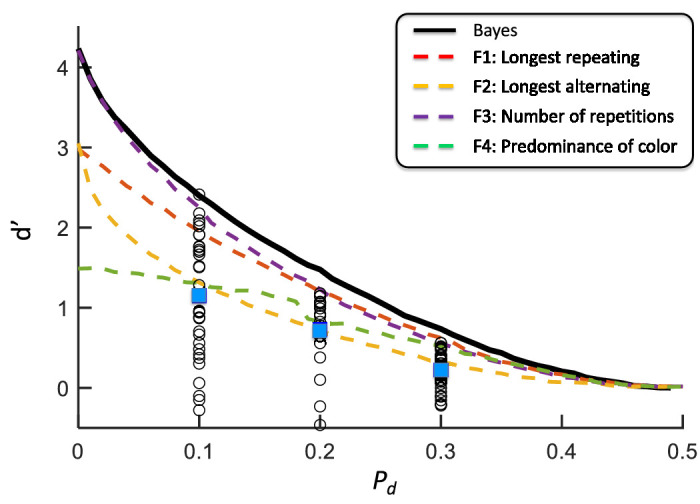
Model performance versus actual. The signal detection theory (SDT) sensitivity parameter *d′* is plotted versus *p_d_*, the probability of disruption. The results for each observer in the three conditions—low disruption, medium disruption, and high disruption—are plotted with empty circles. The mean for each observer in each condition is plotted as blue squares. The observers‘ performance was significantly less than that predicted by the full model (solid black contour). See text. The predictions for the single feature models are plotted as dashed contours in different colors. The contours were computed by Monte Carlo simulation. Each computed value was based on 5,000 Monte Carlo replications. See text.

Our conditions of LD, MD, and HD differed in the degree of disruption *p_d_* = 0.1, 0.2, and 0.3 applied to sequences generated by a Markov Generator with Markov parameter *p_r_* = 0.9*.* In Figure 4^5^, we plot sensitivity *d′* versus *p_d_* for each observer (white circles) and mark, with a blue square, the mean *d′* of the observers in each condition plotted versus *p_d_*. The value of sensitivity *d′* was estimated using the equal variance Gaussian signal detection model (Figure 1A) (Green & Swets, 1966/1974). The heavy black contour is the expected performance (*d′*) of the full BDT model, the maximum possible performance. The dashed contours are the expected performances for the four of the feature models based on single features shown in Figure 3. The performance of one of the features (F3: number of repetitions) is only slightly below that of the full model. A human observer who used only this feature would do almost as well as the full BDT model.

**Figure 5.**
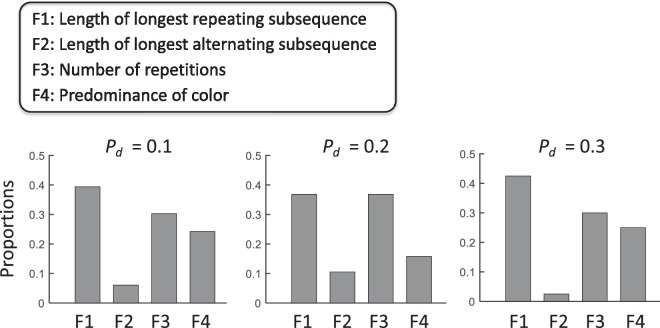
Dominant features. The dominant feature was taken to be the feature with the largest general linear model (GLM) coefficient for each observer. The bar chart summarizes the number of times each feature was ranked as dominant.

This feature was not the feature mentioned most often in observers’ debriefing (see Figure S01 in the Supplement). This feature, F3: number of repetitions, is statistically equivalent to the feature, F3′: number of alternations, because the sum of the number of repetitions and the number of alternations in a binary sequence of length 20 must be 19: the two features have a perfect negative correlation. In debriefing, observers mentioned both features and we coded both as, F3: number of repetitions, for convenience.

### The full BDT model

The observers’ overall performance differed across the three conditions (one-way analysis of variance on *p_d_* and *d′*), F(1,182) = 42.436, *p* < 0.0001. It was significantly greater than 0. Indeed, 92 out of 92 *d′* values are all greater than 0 and 91 out of 92 *d′* are less than the prediction of the full model.

The full BDT model, based on Bayesian decision theory, is not a good match to observers’ performances. Indeed, only one of the 92 observers has performance at or exceeding that of the full BDT model.

### Single-feature BDT models

Next we compared human performance with single feature models for each of the features defined in Figure 3. To quantify which features individual observers relied upon, we standardized regression coefficients using a method proposed by Menard (2004). Menard referred to the resulting weights as fully standardized regression coefficients (his Equation 5). A one standard deviation change in an independent variable induces a change of magnitude equal to the corresponding fully standardized regression coefficient in the dependent variable *logit(Y).*

We identified the feature for each observer and condition that had the greatest standardized coefficient for that observer. We refer to this feature as the observer's dominant feature. We histogram the frequency with which each of the four features was chosen as dominant. Across three conditions, the percentages of the observers for which each feature was dominant were 40.2%, 22.8%, 31.5%, and 5.5% for F1 (the length of the longest repeating subsequence), F2 (the length of the longest alternating subsequence), F3 (the number of repetitions), and F4 (the predominance of color), respectively. F1 and F3 (the number of repetitions) were most frequently selected as dominant features, F1 more often than F3, the feature with highest values of *d′* across conditions (Figure 5).

**Figure 6.**
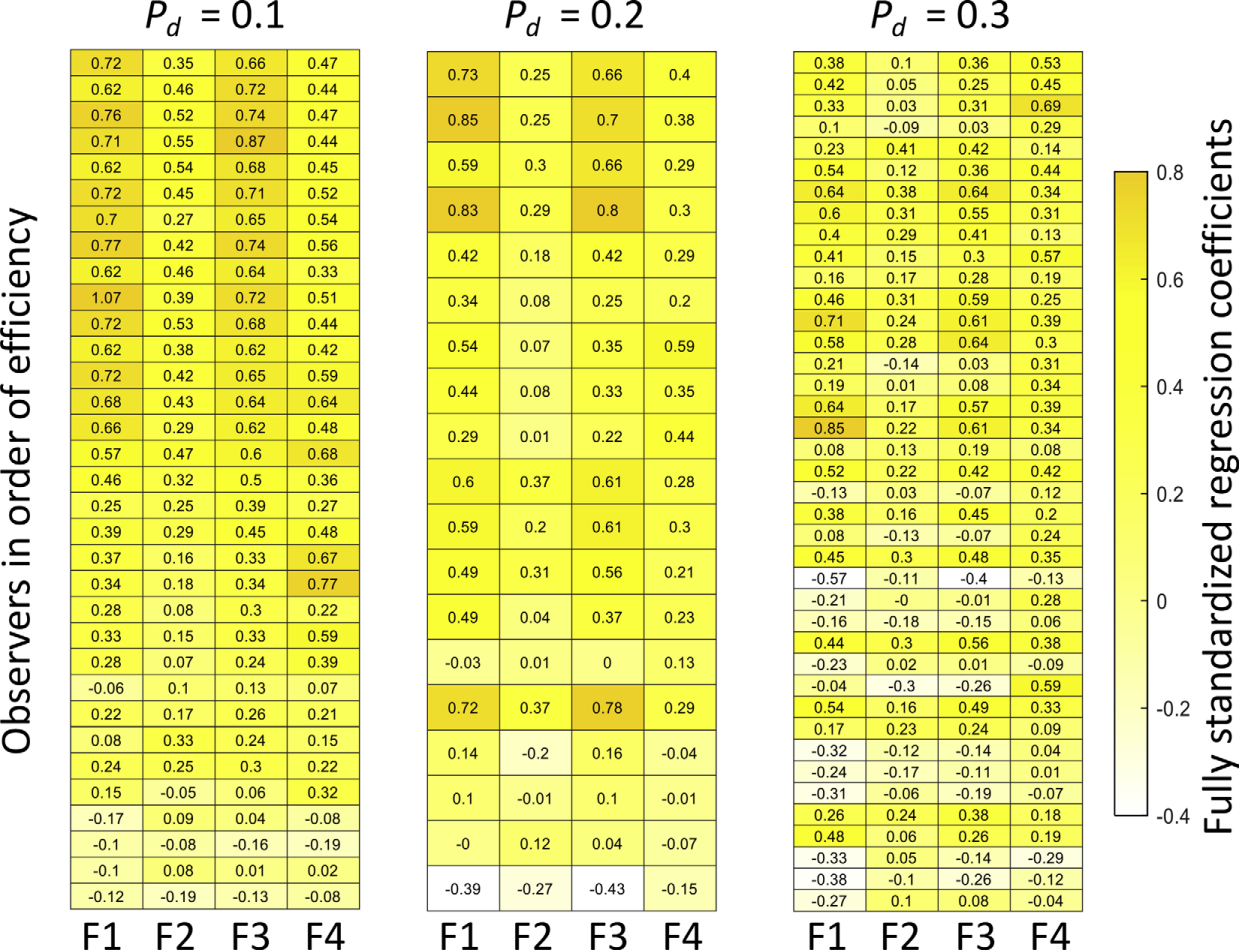
Single-feature models: Individual observers. We considered the possibility that observers did not use the full sequence but rather based their judgments on one or more features of the sequence. The ideal feature observer compares the feature value to a fixed threshold and responds “patterned” or “random,” depending on the results of the comparison. We used logistic regression (Equations 4 and 5) to assess which features—considered in isolation—contributed to observers’ judgment. We plot a color map of fully standardized logistic regression coefficients (Menard, 2004) of the single feature models. A general linear model (GLM) with a logistic link function is performed for each feature, each observer, and each disruption condition. Each row represents the normalized values of the regression. The order of the observers is based on the efficiency measurement.

Next, we examine fits of models based on subsets of features. Did observers rely on only a single feature or did they make use of multiple features? If so, which ones? We used GLM to regress the observer's responses on the four features and then standardized the regression weights using, as mentioned before, a method due to Menard (2004). Figure 6 is a color map of these standardized logistic regression coefficients (Menard, 2004) for each observer in each condition. Each row summarizes the fully standardized weights of the four features for a single observer in one of the three conditions. The rows are ordered by a measure of performance efficiency, highest to lowest, within condition. Efficiency for any observer in any experimental condition is just the ratio of the observer's *d′* value in that condition to the *d′* value of the full BDT model in the same condition.

Larger standardized regression weights are plotted with more saturated shades of yellow. The patterns are striking. For higher efficiency observers in condition 1, the columns corresponding with F1 and F3 are darker, suggesting that the high efficiency is achieved primarily through use of F1 and F3. For less efficient observers, there is little indication that the observers used primarily one or two features. Similarly, in the most difficult condition (*p_d_* = 0.3) observers do not seem to depend on some features to the exclusion of others.

To summarize, observers who achieve high efficiency base their judgments primarily on F1 (length of longest repeating subsequence) and F3 (number of repetitions). The feature mentioned most often by observers is indeed F1 but the second most frequently mentioned feature is F4 (color imbalance), not F3.

### Multifeature BDT models

Can human performance be better captured by a model based on more than one feature? To address this question, we constructed *n*-feature models based on the four features in Figure 3 and all possible subsets of these four features. We denote each model by listing the indices of the features in Figure 3 that are included in it; for example, 124 is the model based on F1, F2, and F4.

**Figure 7.**
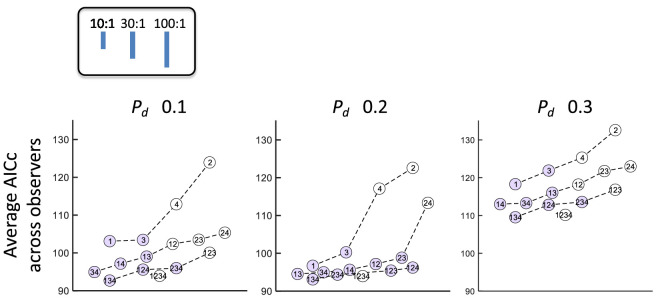
Multifeature model comparison: Fitting performance of multifeature model. We constructed general linear models (GLMs) for all combinations from four or fewer features. The *y*-axis denotes the Akaike information criterion corrected for small sample size (AICc). A smaller AICc indicates a better model fit. Small vertical lines in a legend show the evidence ratio (10:1, 30:1, 100:1) corresponding with the vertical distance between one model's AICc and another model's. Jeffreys (1998) suggests that one model is strongly supported when the evidence ratio is larger than 10. Similarly, evidence ratio of larger than 30 indicates very strong evidence and that of larger than 100 indicates decisive evidence.

We fit 15 GLMs based on 1 or more features for each observer. There are four possible GLMs based on a single feature, six based on two features, four based on three features, and just one based on all four features. For model comparison, we used the Akaike information criterion (AIC) based on the maximum log-likelihood fits, corrected for small sample sizes (AICc). The AICc is a measure of the badness of fit of each model that includes a penalty for the difference in the number of free parameters and the difference in sample size between any two models compared (Burnham & Anderson, 2002). In Figure 7, circles denote models and the vertical coordinate of the circle is the AICc averaged across observers.

Based on the difference in AICc between a model pair, we can compute the evidence ratio as exp(Δ AICc 2). This measure is the relative likelihood of model pairs and represents the evidence that one fitted model is to be preferred to another in a Kullback–Leibler information sense than the other (Burnham & Anderson, 2002).

Jeffreys (1998) suggests that an evidence ratio of greater than 3 represents substantial evidence for one model against the other model. We used an evidence ratio of greater than 10 (strong evidence in Jeffreys’ terms) as a more conservative criterion. This criterion is plotted as a vertical line segment in an embedded legend. We also include line segments corresponding to 30:1 and 100:1 criteria. The models that are closer than the 10:1 criterion to the best fitted model in each condition are marked in purple whereas the models above the criterion are marked in white.

### Multimodel inference

Burnham and Anderson (2002) recommend a multimodel approach in which we use the information in Figure 7 to eliminate features that have little support and find combinations of features that best account for the data. In single feature models, we found that the length of the longest repeating subsequence and the number of repetitions dominate the two other features regardless of the probability of disruption. Among two-feature models, 12, 13, and 23, are best supported by data in the LD and HD conditions. In the condition MD, the two-feature models 14 and 24 were also equally supported. Given these results, unsurprisingly, 123 is the best supported among three-feature models in all conditions.

In terms of model parsimony, to what extent the model can be improved by adding one more variable is important. The evidence ratios between the best single feature model and the best two-feature model for the LD, MD, and HD conditions were 59.2, 3.0, and 12.8, respectively, whereas the evidence ratios between the best two-feature model and the best three-feature model were 3.1, 2.0, and 5.4, respectively. At least two features are needed to adequately fit the data. Adding one more feature to the two-feature model does not substantially increase the model fitting performance. We tentatively conclude that observers overall are using two features of the four we consider. To summarize, F1 (the longest repeating subsequence) and F3 (number of repetitions) are the critical features to capture human performance in discriminating patterns.

## Discussion

We measured human performance in a signal detection task where observers were asked to judge whether binary texture sequences of blue and yellow tokens had been generated as independent, identically distributed realizations of a binary Bernoulli random variable with probability 0.5 of blue (a fair coin), or as a Markov chain whose output had been disrupted by flipping some of the tokens with probability *p_d_*. In three conditions, *p_d_* was set to 0.1, 0.2, or 0.3.

We first compared human performance to that of a BDT model (the full BDT model) that maximized the expected number of correct classifications (see the Appendix). The performance of the Full BDT model was significantly better than that of the human observers and we could reject the full BDT model in all conditions.

We next considered the possibility that observers basing their judgment on a BDT analysis, but using only part of the information available to them in the form of pattern features. A pattern feature was defined to be an integer-valued function of a sequence, such as the length of the longest repeating subsequence in the sequence. Intuitively, a pattern feature is a measure of a particular deviation from randomness (Bernoulli).

We developed BDT models based on single features (Figure 3) and compared the *d′* values for each single feature model to that of the full model. These features were the features observers spontaneously claimed to be using (Supplement). We then matched human performance to that based on single features and found no satisfactory match for any of the single feature models considered when their performance was compared to models based on multiple features (Figure 7). The pattern of results in Figure 6 for the more efficient observers in LD indicate that many observers are using more than a single feature. The pattern is present in MD, but not obviously in HD.

We further compared human performance to that of a multifeature BDT model—models based on more than one feature—using a model comparison approach based on the AICc (Burnham & Anderson, 2002). We found acceptable models of human performance based on as few as two features.

We also found evidence that human performance predominantly uses a limited subset of pattern features, as few as two. We show that we can objectively identify some of these features and test hypotheses about how they are combined for the signal detection task we used. The selection of features we identified and evaluated give us a tentative pool to start with, but more features are likely needed to capture human performance in a wider variety of tasks. At least some of the observers in some of the conditions are using more than one of the features we considered.

It is possible that there are other features used by observers which would better match human performance alone or in combination with the features we did consider. We only considered weighted linear combinations of features (GLM, Equations 4 and 5), and these features were those volunteered by the observers themselves (Supplement). It is also plausible that models permitting nonlinear combinations of features would better match human performance. The use of multiple features that each capture partial information about a sequence is not surprising given the evident complexity of the Bayesian computation (Appendix) and parallel work in depth cue combination (Landy et al., 1995; Maloney & Landy, 1989).

### Sequential tasks

In the task considered here, observers saw the entire sequence all at once and it remained open to inspection until they responded. We could consider alternative tasks where the observer is shown the tokens in a sequence one at a time only briefly. For example, the sequence of successes and failures for a basketball player or the successive outcomes of a roulette wheel. Detecting patterns and predicting in such sequences is only possible if observers retain information about past outcomes. The methods we develop here could equally be applied to such sequential presentation tasks to investigate errors in judgment associated with the hot hand or the gambler's fallacy and determine which features are retained.

### The pattern feature pool model

**Figure 8.**
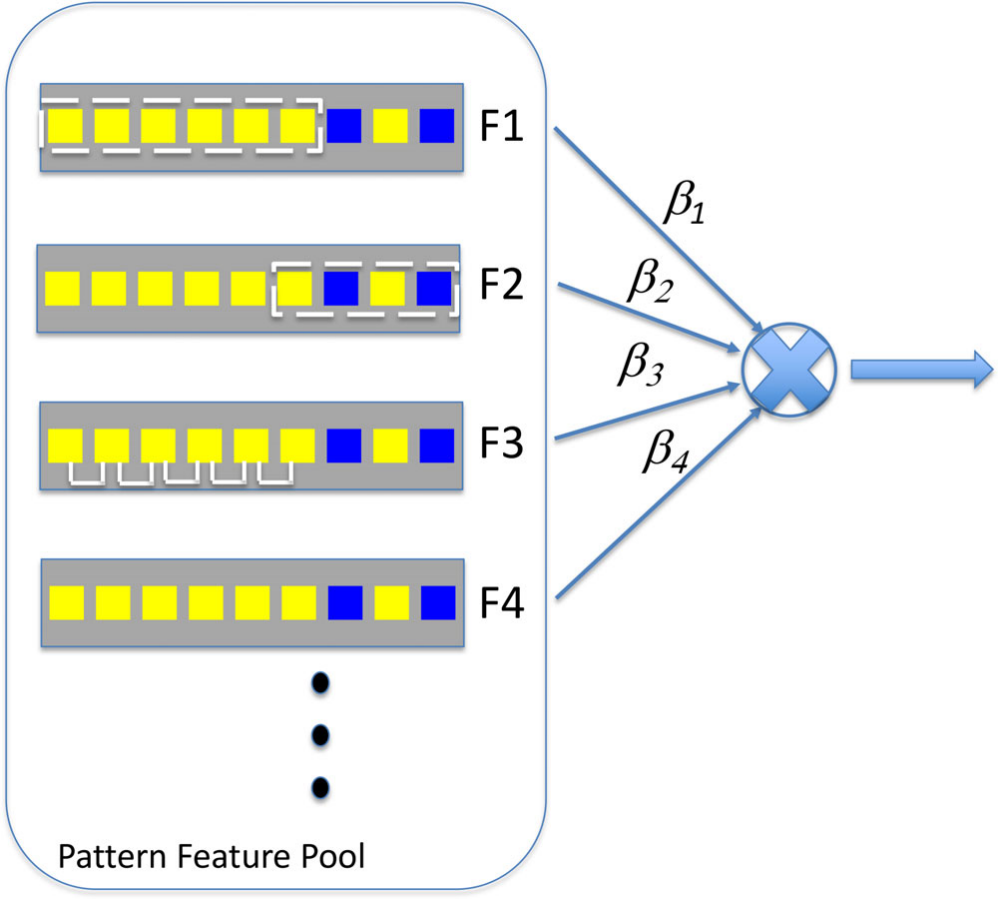
Pattern feature pool model. The pool comprises features for a binary sequence. The sequence in the illustration is the sequence of Figure 3, truncated to improve legibility. The four features shown are also taken from Figure 3, and we indicate the possibility that there may be additional features. A first goal for research is to identify which features are present in the pool. The values of these features are weighted and combined and a second goal for research is to identify the rule of combination.

We consider the possibility that in a wider range of pattern tasks human observers have access to a pool of pattern features and flexibly combine these features to carry out the tasks (Figure 8). In Figure 8, the information from the features is weighted and then combined. The observer may have used different pool features with different weights for different tasks. The weighted linear rule of combination we used in this article is specified in Equation 4 and Equation 5. The *k^th^* feature is weighted by a weight *β_k_* and then the weighted values are summed.

This sort of model was commonplace in early work on pattern recognition (Uhr, 1966; Uhr & Vossler, 1966) with Selfridge's pandemonium (Selfridge, 1966) as perhaps the best known example. Uhr and Vossler developed a learning model that used 5 × 5 feature templates as input to a classifier that allowed recognition of hand-written letters. Their classification algorithm evaluated the recent contribution of each template feature and could replace template features that were contributing little by new template features chosen at random or by design. Eventually the classification algorithm would settle on a stable set of features that were combined to classify letters. Our model is close in spirit to that of Uhr and Vossler.

Could a fixed set of pattern features (a feature pool) account for human performance in a wider variety of tasks concerned with detecting deviations of randomness? We consider some candidate tasks.

### Human generation of random sequences

Reichenbach (1949) asserted that human observers could not generate sequences that would pass standard tests of randomness. Donald Knuth in a section of his *Art of Programming* devoted to pseudo-random number generators and tests of randomness amplified Reichenbach's point: “If we were to give some man a pencil and paper and ask him to write down 100 random decimal digits, chances are very slim that he will give a satisfactory result. People tend to avoid things which seem nonrandom, such as pairs of equal adjacent digits…. And if we were to show someone a table of truly random digits, he would quite probably tell us they are not random at all; his eye would spot certain apparent regularities” (Knuth, 1971, p. 34ff). Knuth is outlining a model of generation based on avoiding certain features (“apparent regularities”), those associated with various kinds of patterns.

We can imagine an observer constructing a random sequence while continually monitoring the token-by-token values of a set of features (e.g., those in Figure 3), each of which signals a potential failure of randomness in a graded fashion. The observer might hesitate between extending a binary sequence of blue and yellow tokens by appending a yellow token or a blue token and pick whichever extension—blue or yellow—is judged more random.

### Illusory patterns

Skinner (1942) examined tendencies to alternate and repeat in generating random sequences and finds deviations from what we might expect of a fair coin. See (Wagenaar, 1970a; Wagenaar, 1970b; Wagenaar, 1972) and Neuringer (1986) for surveys of an extensive literature.

Maloney et al. (2005) asked observers to view a bistable motion display (motion quartet) and classify the direction of rotational movement as clockwise *C* or counterclockwise $\bar{C}$. The motion seen and its direction were illusory with clockwise and counterclockwise equally likely overall. However, observers tended to see sequences of repetitions such as *CCCC* or $\bar{CCCC}$ or sequences of alternations $\bar{C}C\bar{C}C$ or $C\bar{C}C\bar{C}$ more often than would be expected if successive trials were statistically independent. Given ambiguous stimuli, observers effectively hallucinated repeating and alternating patterns. We conjecture that we could use analogous methods to the methods we use here to identify the regularities people avoid and compare them with the features we identify here.

### Features, heuristics, and bags of tricks

Is the pattern feature pool model proposed here just a kind of heuristic (Gigerenzer, 2000; Gigerenzer & Gaissmaier, 2011; Gigerenzer et al., 1999)? The definition of heuristic (e.g., Gigerenzer & Gaissmaier, 2011; Tversky & Kahneman, 1974) is contentious, but a heuristic would seem to be—above all—a rule that makes a decision or selects an action. The heuristic is in competition with an optimal rule and may be chosen over the optimal rule because it is faster, easier to execute, or otherwise superior to the optimal rule. Heuristics are reminiscent of bag-of-tricks approaches in modeling perception (Cornelissen, Brenner, & Smeets, 2003; Ramachandran, 1985; Ramachandran, 1990) where visual phenomena are explained by simple rules. There is no obvious rule for combining tricks when more than one trick is applicable.

We do not want to enter into this controversy, except to contrast two differences between the use of heuristics/tricks and the feature combination model emerging here. Heuristics and bags of tricks—as we understand them—are rules that make decisions or select actions for us. Features are, in contrast, measures of evidence used to make decisions and they may readily be combined (Landy et al., 1995). Given two or more heuristics or tricks from the bag, it is unclear which to make use of, what the criteria for use are, or how to combine them.

One attraction of such pattern feature pool models is that they break a difficult task into two stages: select simple features that are readily and unambiguously computable (the feature pool) and then carry out the task by assigning weights to each feature and combining them. A second is that (as suggested by Uhr and Vossler) new features can be added to the pool to improve performance and old features that contribute little to performance can be dropped. One may evolve a powerful feature pool gradually. A third is that many judgments concerning randomness and pattern can share this common feature pool.

## Supplementary Material

Supplement 1

## Appendix Appendix

### The decision rule for the full Bayesian model

Let *S* = *S*_1_*S*_2_…*S_n_* denote the sequence of blue and yellow tokens with *S_i_* taking values 0 (yellow) or 1 (blue) and *n* = 20. There were two generating processes for sequences, RANDOM and DMS. On each trial one of the two generators was chosen at random with equal probability. We use Bayes theorem in odds form to compute the posterior odds in favor of DMS

(A1) $$\begin{array}{ccc} \lambda \; & = & \frac{P[DMS|S]}{P[RANDOM|S]}\\ & = & \frac{P[S|DMS]}{P[S|RANDOM]}\frac{P[DMS]}{P[\mathrm{RANDOM}]}\; \end{array}$$

and, as the last term (the prior odds) is 1,

(A2) $$\begin{array}{c} \lambda =\frac{P[S|DMS]}{P[S|RANDOM]}.\; \end{array}$$

The term *P*[*S* ∣ *RANDOM*] is just 2^–^*^n^* and we rewrite Equation (A2) as

(A3) $$\begin{array}{c} \lambda =2^{n}P\left[S|DMS\right].\; \end{array}$$

The normative full model selects the response “patterned“ DMS if λ > 1: in effect, it selects whichever of the two generators assigns a higher probability to the sequence observed. To compute Equation (A3), we need to compute *P*[*S* | *DMS*]. We are given the probability of repetition *p_r_* and the probability of disruption *p_d_* that characterize the DMS.

First, we define the disruption vector *D* = *D*_1_*D*_2_⋅⋅⋅*D_n_* to be a vector of 1's and 0's with a 1 in each disrupted location. The sequence length is as before *n* = 20. If we knew the disruption vector of a DMS we could “undisrupt” it and recover the underlying Markov sequence (Figure 2B). Let *M* = *M*_1_*M*_2_⋅⋅⋅*M_n_* denote that (unknown) Markov sequence.

The XOR of two bits (binary digits with values 0 and 1) is 0 if the two bits are the same otherwise 1. The XOR (denoted ⊕) has the interesting property that if C = A ⊕ B, then A ⊕ C = B and B ⊕ C = A. If we apply the operation ⊕ on the vectors *S*, *D*, and *M* we can represent the disruption operation as *S* = *D* ⊕ *M* and recover the Markov Sequence from the DMS by *M* = *S* ⊕ *D*.

To compute the probability of the *S* when the generator is DMS, we must sum the probabilities of all the possible ways it might have come about. The key insight is that, whatever, the output of the Markov chain, there is a disruption vector that would transform it into any target sequence *S*. As an example, let's consider sequences of length *n* = 3 and assume we observe a sequence yellow–blue–yellow coded as 101. Then it could have come about because the Markov chain produced 101 and there were no disruptions. The disruption vector is 000. Or the Markov chain could have produced 001 with disruption vector 100. Or the Markov chain could have produced 111 with disruption vector 010. We must consider all the possible combinations of Markov chain and disruption in computing the probability of the generation of a particular sequence *S* by the DMS:

(A4) $$\begin{array}{c} \;\;\;\;\;P[S|DMS]=\sum P[M|MS]P[S⊕M]\; \end{array}$$

The sum is over all possible patterns of length *n* and *MS* denotes generated by the Markov sequence generator. Each term in the sum is a combination involving a Markov pattern *M* and the unique disruption *D* such that *S* = *M* ⊕ *D*. Each term in the sum is mutually exclusive and we are justified in adding them. For any disruption pattern *D*, the probability *P*[*D*] = *P*[*S*⊕*M*] is determined by the probability of disruption *p_d_*. If σ(*D*) is the number of disruptions in the disruption vector (the sum of the 1s and 0s in the vector), then

(A5) $$\begin{array}{c} P\left[D\right]=p_{d}^{\sigma (D)}{\left(1-p_{d}\right)}^{n-\sigma (D)}.\; \end{array}$$

The last term to compute is the probability *P*[*M* ∣ *MS*(*p_r_*)] that a sequence *M* is generated by a Markov process *MS*(*p_r_*) with probability of repetition *p_r_*. The entries in the sequence *M* = *M*_1_*M*_2_⋅⋅⋅*M_n_* are not statistically independent but we can recode the sequence in terms of its transitions which are independent. Let *V_i_* = *M*_*i* − 1_∧*M_i_* for *i* = 2, ⋅⋅⋅, *n* where ∧ denotes the logical operation AND. *V_i_* is TRUE (1) if the current token is the same color as its predecessor otherwise 0 (FALSE). We recode the sequence *M* = *M*_1_*M*_2_⋅⋅⋅*M_n_* as *M*′ = *M*_1_*V*_2_⋅⋅⋅*V_n_* with *M*_1_ the initial state and the *V_i_* specify how to generate the succeeding states from each preceding state: *V_i_* = 1 (repetition) or *V_i_* = 0 (alternating). These random variables are independent. Let *V* denote the *n* – 1 length binary vector of alternations/repetitions *V*_2_⋅⋅⋅*V_n_* and σ(*V*) the number of 1s in the vector (the number of repetitions). Then

$$\begin{array}{ccc} P[M|MS]\; & = & P\left[M^{'}|MS\right]\\ & = & \frac{1}{2}p_{r}^{\sigma (V)}{\left(1-p_{r}\right)}^{n-1-\sigma (V)} \end{array}$$

and the values of Equations (A3), (A4) and (A5) are sufficient to compute Equation (A2).

### The BDT decision rule for the single-feature models

The decision rule for a single feature model based on the *k^th^* feature is *λ_k_* > 1, where

(A6) $$\begin{array}{c} \lambda_{k}=\frac{P\left[DMS|\varphi_{k}\left(S\right)\right]}{P\left[Random|\varphi_{k}\left(S\right)\right]}\; \end{array}$$

is the posterior odds based on the *k^th^* feature and *L_K_*, a likelihood criterion. Because the prior odds of the two generators is 1,

(A7) $$\begin{array}{ccc} \lambda_{k}\; & = & \frac{P\left[\varphi_{k}\left(S\right)|DMS\right]}{P\left[\varphi_{k}\left(S\right)|\;\mathrm{Random}\right]}\frac{P[DMS]}{P[\mathrm{Random}]}\\ & = & \frac{P\left[\varphi_{k}\left(S\right)|DMS\right]}{P\left[\varphi_{k}\left(S\right)|\;\mathrm{Random}\right]}. \end{array}$$

For any of the features we consider, the feature φ*_k_*(*S*) can take on only a finite number of integer values *v*_1_,*v*_2_,⋅⋅⋅, *v_p_*. For example, the longest repeating subsequence in a sequence of length 20 must be an integer between 1 and 20. We can compute *P*[φ_*k*_(*S*) = *v* ∣ *DMS*] by Monte Carlo simulation. We generate a large number of DMS sequences and observe what proportion have φ_*k*_(*S*) = *v* for each value of *v*. This proportion approximates *P*[φ_*k*_(*S*) ∣ *DMS*].
