# Supplementary material for: Detecting visual texture patterns in binary sequences through pattern features
Source: J Vis. 2023 Nov 1;23(13):1. doi: 10.1167/jov.23.13.1 (PMC10627294; doi:10.1167/jov.23.13.1)
Supplement: Supplement 1 [file jovi-23-13-1_s001.docx]

**Supplement to**

Detecting Visual Texture Patterns in Binary Sequences through Pattern Features

Maria F. Dal Martello Keiji Ota

Dana E. Pietralla Laurence T. Maloney

**1. Features Reported by Observers**

After the last trial of the experiment each observer was asked to answer the following question *“What made you think the sequence was either random or non-random?”.* The experiment was conducted in Italian and we translate all questions and answers that we discuss here from Italian to English. The term “difetto” was used in describing the disruption process to observers. We translate it as “defect” here. Original data in Italian is available from the authors.

Observers’ responses were categorized as either one of five features or “Other”. The response *“if there were more than 4 squares of the same color together I thought the sequence wasn’t random*”, for example, was coded as feature F1, *length of longest repeating subsequence.* Recall that for our purposes a feature is any numerical function of a sequence.

Several observers reported more than one feature, few did not report any. Responses that could not be interpreted as a feature (e.g. “*Harmonious connections between yellow and blue*”) were classified as “Other”. About one third of responses were classified as “Other”.

FEATURES REPORTED BY OBSERVERS

**Figure S01, percentage of each kind of reported features on total reported features for each condition.** The conditions differ in the probability of disruption: LD (*p_d_ =* 0.1), MD (*p_d_* = 0.2), HD (*p_d_*  = 0.3). We coded as "Other" features that could not be otherwise classified. Features F3 and F3' are redundant since the number of repetitions plus the number of alternations for any binary sequence of length 20 is always 19. We report here how subjects chose to describe this feature but in the main text we use only ”number of repetitions”.

**Observers’ Responses by Condition**

**Condition LD (Low Disruption, *p_d_* = 0.1)**

01: Rows of squares of the *same color* and *regularity.* [F1]

02: I considered sequences with length of *same color* squares *≥ 15*. [F1]

03: *Same color* groups size. [F1]

04: Higher the number of *same color* *squares in succession*, less random the sequence is. [F1]

05: I looked for *logical connections* and precise sequences *despite the defects.* [Other]

06: I followed the *training*, I looked for sequences. [F1], [Other]

07: *Length of strings of same color* squares. [F1]

08: The *length of strings of same color.* [F1]

09: Number of squares of the *same color* *in succession.* [F1]

10: *More than 5 squares of the same color* in succession. [F1]

11: A*t a glance*, if it didn’t help I applied the rule. [Other]

12: Estimate of the *probability* of the following squares. [Other]

13: I think it wasn’t random. [Other]

14: Tiredness. [Other]

15: *Length of subsequence of the same color.*  [F1]

16: Many *squares of the same color next to each other* and a logic in the sequence made me think the sequence wasn’t random; lack of logical rules and *mixed colors* made me think it was a random sequence. [F1], [F2], [Other]

17: The *number of both blue and yellow squares* and a *comparison* of this number between one sequence and the next. [F4]

18: First I considered the *number of repetitions*, then I tried to see if the sequence *matched the outcomes of a series of coin tosses.* [F3], [Other]

19: Non-random if the sequence begun and ended with a *logical order*, if I couldn’t see any rule I thought it was random. [Other]

20: The perception of a *mathematical logic* or, irrationally, if a *color was almost the only one* present. [F4]

21: I considered to be random a sequence with *variable distribution* and I considered a sequence to be non-random if the series of same color squares were *predominantly either yellow or blue.* [F4]

22: A *logical connection* in the sequence. [Other]

23: *At a glance*, *ratio between yellow and blue squares number.* [F4]

24: N.C. [illegible]

25: I thought that *alternating colors* were cues of random sequences. [F3']

26: Presence of *a series of same color*  in the sequence. [F1]

27: The *predominance of one color* or a *rule* in same color series. [F4]

28: Long series of *repetitions.* [F1]

29: First I considered the *difference between the number of squares of each color*, if the *difference* was *big* I thought the sequence was *random*; second I considered the number of repetitions and if there were *more than 4 squares of the same colo*r together I thought the sequence wasn’t random. [F1], [F4]

30: Number of *successive* *repetitions of the same color*; *symmetry between yellow and blue* squares; their *order*; total *number of each color*. [F1], [F4]

31: Many repetitions made me think it was a *random* sequence. [Other]

32: *Alternation* and *difference in the number of each color* squares. [F3'], [F4]

33: If there were around *10 squares of each color I thought the sequence was random.* [F4]

**Condition MD (Medium Disruption, *p_d_* = 0.2)**

01: *Longer strings of one of the colors; predominace of one color.* [F1], [F4]

02: The *predominance of one color* with short interruptions by the other color. [F1], [F4]

03: *Many repetitions* of the *same color* with around 3% of probability. [F1]

04: If the *interruption of* a series of *same color* was *just one different color*, I thought it was a *pattern;* if there were *many same color squares in a row* I thought it was a pattern; if the sequence was *varying* a lot I thought it was random. These *rules* were *vague*; soon the task became senseless. [F1], [F3']

05: The *number of* *detected disturbing squares.* [Other]

06: *Strings of yellow squares*; *alternations* of yellow and blue following a rule. [Other]

07: *Alternating strings of same color*. [F2]

08: I thought the sequence was random if there were *strings of 10 or more squares alternating.* [F2]

09: The number of *defects.* [Other]

10: *Blue squares in a logical arrangement.* [Other]

11: Number of *same color squares* next to each others. [F1]

12: Number of *same color squares* in succession and number of squares interrupting the succession. [F1]

13: *Long strings of same color* indicate patterns. [F1]

14: Longer the *strings of the same color* more likely the sequence isn’t random. If the sequences of the same color were short (1 -2 squares) I thought it was random. [F1]

15: No answer.

16: *If changing the color of a few squares I obtained a symmetric sequence* I decided it wasn’t random. [Other]

17: Presence of *rules.* [Other]

18: If there were *repeated schemas* or *possible schemas disrupted* by noise or many squares of the *same color in succession* I thought it wasn’t random; if I was uncertain I acted by *instinct.* [F1], [Other]

19: More squares of the *same color in succession*, more likely it wasn’t random. [F1]

**Condition HD (High Disruption, *p_d_* = 0.3)**

01: I thought the sequence was random if there were excessive *alternations* of colors. [F2], [F3']

02: When *2 or more squares of the same color* were *next to squares of different color*, I thought they were *not defects* in a pattern, but *part of a random sequence.* [F3']

03: Since it’s more likely that the colors distribution is more heterogeneous in a random sequence, colors *alternating* more frequently indicated a random sequence. [F3']

04: I considered the number of *same color squares in succession.* [F1]

05: The number of *same color squares in succession* and *reasonable and harmonious interruptions.* [F1], [Other]

06: More of *same color squares in succession*, more the sequence gave me the idea of a pattern. [F1]

07: The number of *same color squares in succession.* [F1]

08: Number of *same color squares in succession.* [F1]

09: The *regularity in the sequence*, patterns with defects have been the most difficult to detect. [Other]

10: Mostly I relied on the impression *at a glance.* [Other]

11: *Repetition of schemas* in the structures. [Other]

12: A *rough impression* without a precise rule. [Other]

13: *Order i*n the sequence. [Other]

14: Number of *same color squares in succession.* [F1]

15: At the beginning I considered the *probability of blue or yellow following blue*, then I considered *regularity.* [Other]

16: If the number of the *same color squares* in *succession* was *≥ 9* I thought it was a pattern, I considered also *alternating series of same lenght and color.* [F1], [Other]

17: Sometimes I followed the feeling, sometimes I reasoned. [Other]

18: I followed the *intuition system* (Kahneman, 2003). [Other]

19*: Logical series.* [Other]

20: *Repetitions.* [F3]

21: *Number of each color squares*, if the number was the same for blue and yellow the sequence was random, bigger the difference between the color's number less likely that the sequence was random. [F4]

22: Groups of *same color* squares. [F1]

23: No answer.

24: The number of squares of *different color* (2 yellow and 1 blue=> pattern). [F4]

25: The *number of each color* squares. [F4]

26: A big difference between the *number of yellow and blue* squares (non random) in some sequences; a feeling there were patterns; long blocks of *same color* squares looking as non random. [F1], [F4], [Other]

27: *Symmetry*; clear cues of randomness or not. [Other]

28: 1. If the number of one set of same color squares was clearly different from the number of the sets of the different color (e.g. 5-6 vs 15-14) I concluded the sequence wasn't casual. 2. If there were *repetitions of alternating mini-sets* of same color (e.g. 3 blues in a row, 3 yellows in a row, etc.), I concluded the sequence wasn’t random. [F4], [Other]

29: *Geometry*, combination possibility. [Other]

30: *Harmonious connections* between yellow and blue. [Other]

31: Too long strings of same color squares. [F1], [Other]

32: *At a glance.* [Other]

33: The *yellow/blue* *ratio* and number of *same color* squares next to each other. [F1], [F4]

34: I divided the sequence in quarters, calculated *how many yellow and blue* and if there was an imbalance in more than 2 quarters I said "non random". [F4]

35: Some strange *logic, taste for order*, tiredness, exhaustion, sometimes an interior certainty. [Other]

36: *Intuition* and too many squares of same color made me suspicious. [F4], [Other]

37: *Intuition* at the beginning; then I based my judgment on the *different number* of squares for each

color. [F4], [Other]

38: The order of presentation. [Other]

39: I tried to estimate how realistic was a series of *same color* squares; I was affected by my previous judgment and I went by *intuition* even if not conforming to the training. [F1], [Other]

40: The *number of squares* for each color. [F4]
